# Titanium particle-induced osteogenic inhibition and bone destruction are mediated by the GSK-3*β/β*-catenin signal pathway

**DOI:** 10.1038/cddis.2017.275

**Published:** 2017-06-15

**Authors:** Ye Gu, Zhirong Wang, Jiawei Shi, Liangliang Wang, Zhenyang Hou, Xiaobin Guo, Yunxia Tao, Xiexing Wu, Wei Zhou, Yu Liu, Wen Zhang, Yaozeng Xu, Huilin Yang, Feng Xue, Dechun Geng

**Affiliations:** 1 Department of Orthopedics, The First Affiliated Hospital of Soochow University, Suzhou, China; 2 Department of Orthopedics, Soochow University Affiliated First People's Hospital of Changshou City, Changshu, China; 3 Department of Orthopedics, Zhangjiagang Hospital of Traditional Chinese Medicine, Zhangjiagang, China; 4 Orthopedic Institute, Soochow University, Suzhou, China

## Abstract

Wear debris-induced osteogenic inhibition and bone destruction are critical in the initiation of peri-prosthetic osteolysis. However, the molecular mechanism underlying this phenomenon is poorly understood. In this study, we analyzed the involvement of the GSK-3*β/β*-catenin signal pathway, which is important for bone formation in this pathological condition. We established a titanium (Ti) particle-stressed murine MC3T3-E1 cell culture system and calvariae osteolysis model to test the hypothesis that Ti particle-induced osteogenic inhibition and bone destruction are mediated by the GSK-3*β/β*-catenin signal pathway. Our findings showed that Ti particles reduced osteogenic differentiation induced by osteogenesis-related gene expression, alkaline phosphatase activity and matrix mineralization, as well as pSer9-GSK-3*β* expression and *β*-catenin signal activity. Downregulation of GSK-3*β* activity attenuated Ti particle-induced osteogenic inhibition, whereas the *β*-catenin inhibitor reversed this protective effect. Moreover, the GSK-3*β/β*-catenin signal pathway mediated the upregulation of RANKL and downregulation of OPG in Ti particle-stressed MC3T3-E1 cells. In addition, our *in vivo* results showed that Ti particles induced bone loss via regulating GSK-3*β* and *β*-catenin signals. Based on these results, we concluded that the GSK-3*β/β*-catenin signal pathway mediates the adverse effects of Ti particles on osteoblast differentiation and bone destruction, and can be used as a potential therapeutic target for the treatment of peri-prosthetic osteolysis.

Peri-prosthetic osteolysis and subsequent aseptic loosening are the most common and potentially devastating long-term complications of total joint arthroplasty.^1^ This pathologic phenomenon results from the biological responses of macrophages, osteoclasts and osteoblasts to wear debris generated from the articulating surface of a prosthesis.^2, 3, 4, 5, 6, 7, 8, 9, 10^ Although macrophages and osteoclasts are integral molecules underlying bone destruction in peri-prosthetic osteolysis, recent studies demonstrated that wear debris also contributes to osteolysis by impairing the osteogenic potential of osteoprogenitors.^4, 5, 6, 7, 8^ Given that osteolysis depends on a balance between osteoblast bone formation and osteoclast bone resorption, an inhibitory effect on osteoblast production can accelerate bone loss. However, the molecular mechanisms underlying the particle-induced osteogenic inhibition are poorly understood.

Glycogen synthase kinase-3 (GSK-3), a serine/threonine kinase, is a critical regulator of glycogen metabolism. GSK-3 has two isoforms, GSK-3*α* and GSK-3*β*, which share 97% amino-acid sequence similarity within their kinase catalytic domains.^11^ These proteins regulate multiple biological processes, including bone formation and remodeling during growth and development. Of the two isoforms, GSK-3*β* is more important for bone remodeling, as demonstrated by increased bone formation in GSK-3*β*^+/–^ mice.^12^ GSK-3*β* is a member of the *β*-catenin destruction system (APC and axin), which initiates the degradation of *β*-catenin in the absence of Wnt signals. Furthermore, inactivation of GSK-3*β* decreases the phosphorylation of *β*-catenin and prevents the degradation of *β*-catenin by proteasome. Stabilized *β*-catenin accumulates and translocates to the nucleus, where it binds the TCF/LEF family of transcription factors that subsequently activate specific genes.^13, 14^ It is known that *β*-catenin is critical for Wnt/*β*-catenin pathway activation, which is essential for proper bone development, and inhibition of this signal pathway downregulates bone formation.^14^ Clinical results using pharmacological antagonists of GSK-3*β* showed that the Wnt/*β*-catenin signal increased bone stock via the *β*-catenin signal pathway.^15^ Our previous studies have demonstrated that during wear debris-induced osteolysis, GSK-3*β* is constitutively expressed in the form of phosphor-Ser-9-GSK-3*β* at a low level, but not GSK-3*β*.^16^ However, to our knowledge, studies have not reported the role of GSK-3*β/β*-catenin signals in osteoblast differentiation and bone destruction during aseptic loosening initiated by wear debris.

Therefore, in this study, we hypothesized that wear particles may reduce osteoblast function and promote bone destruction via the GSK-3*β/β*-catenin signal pathway. We established a murine clavarial model of titanium (Ti) particle-induced bone destruction, along with a murine MC3T3-E1 osteoblast cell culture system to verify this hypothesis.

## Results

### Effects of Ti particles on osteogenic activity

**Figure 1:**
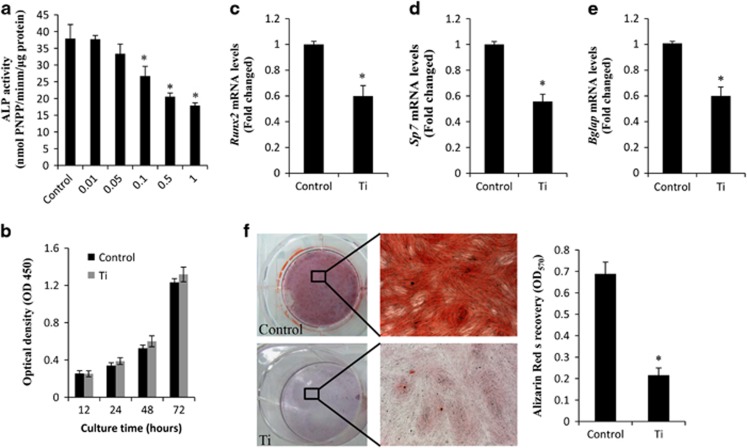
Ti particles inhibiting osteogenic differentiation. (**a**) MC3T3-E1 cells (9 × 10^4^ per well) were incubated in osteogenic medium containing various concentrations of Ti particles (0.01, 0.05, 0.1, 0.5 and 1 mg/ml) for 3 days. ALP activity was determined. (**b**) Cells (10^4^/ml) were cultured with Ti particles (0.1 mg/ml) for 24, 48 or 72 h, and cell viability was determined by CCK-8 assay. mRNA levels of (**c**) *runx2*, (**d**) *sp7* and (**e**) *bgalp* were determined using RT-PCR. (**f**) Matrix mineralization of differentiated MC3T3-E1 cells assessed by ARS staining. *P<0.05 *versus* control

As shown in Figure 1a, Ti particles (doses ⩾0.1 mg/ml) significantly suppressed alkaline phosphatase (ALP) activity when compared with controls. The CCK-8 results showed that treatment with 0.1 mg/ml Ti particles had no toxic effects on cell viability (Figure 1b). Therefore, 0.1 mg/ml Ti particles were used in the following *in vitro* study. RT-PCR results showed that Ti particles (0.1 mg/ml) significantly reduced the gene expression of *runx2*, *sp7* and *bgalp* compared with that in control group (Figures 1c–e). The effect of Ti particles on mineralization was assessed with Alizarin Red S (ARS) staining, and quantified using a colorimetric assay (Figure 1f) after 21 days of treatment. The results of this study showed that Ti particles could profoundly inhibit cell mineralization.

### Ti particles and GSK-3*β/β*-catenin signal pathway

**Figure 2:**
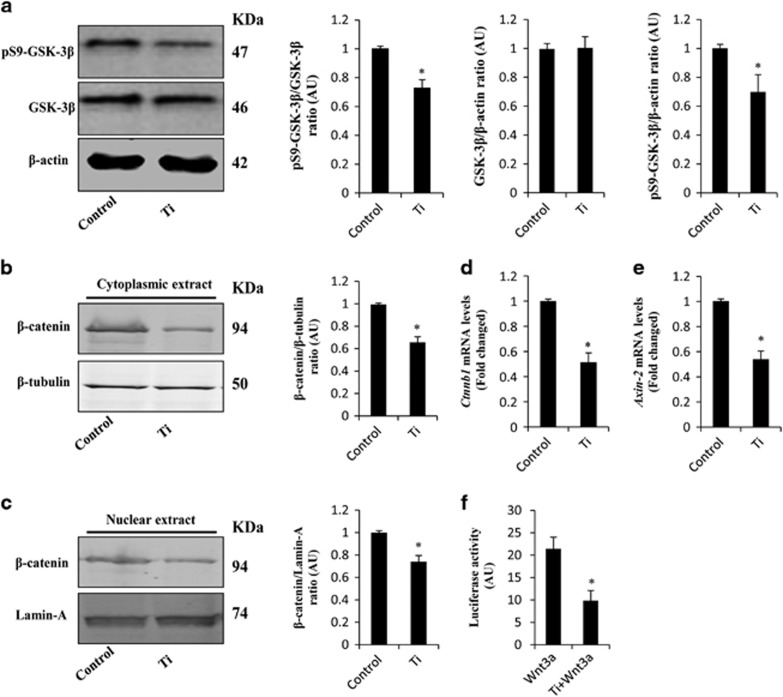
Ti particles regulate the activity of GSK-3*β/β*-catenin signal pathway in MC3T3-E1 cells. MC3T3-E1 cells were cultured in osteogenic medium and 0.1 mg/ml Ti particles for 24 h. (**a**) pSer9-GSK-3*β* and GSK-3*β* were determined by western blot. (**b** and **c**) Cytoplasmic and nuclear extracts obtained from the cells were subjected to western blot analysis using *β*-catenin antibody. (**d** and **e**) Gene copies of *β*-catenin and axin-2 were judged using RT-PCR. (**f**) MC3T3-E1 cells transiently transfected with TopFlash plasmids were stimulated with Ti particles with or without Wnt3a. After 12 h, luciferase activity was measured in cell lysates. *P<0.05 *versus* control

As shown in Figure 2a, after stimulation with Ti particles for 24 h, the expression of pSer9-GSK-3*β* was markedly reduced when compared with control groups. In contrast, Ti particles did not affect the expression of GSK-3*β*, thus indicating that Ti particles could stimulate the activation of GSK-3*β* in MC3T3-E1 cells. Considering the critical role of GSK-3*β* on *β*-catenin, we next determined the expression of *β*-catenin in Ti particle-stimulated MC3T3-E1 cells. Western blot analysis demonstrated that Ti particles decreased *β*-catenin levels in the cytoplasm and nucleus (Figures 2b and c). In addition, RT-PCR results indicated that Ti particles suppressed the mRNA levels of *ctnnb1* and *axis-2*, a *β*-catenin target gene (Figures 2d and e). Furthermore, Topflash reporter assay showed that Ti particles suppressed *β*-catenin-dependent transcription induced by Wnt3a (Figure 2f).

### GSK-3*β/β*-catenin signal pathway and Ti particle-induced osteoblast differentiation

**Figure 3:**
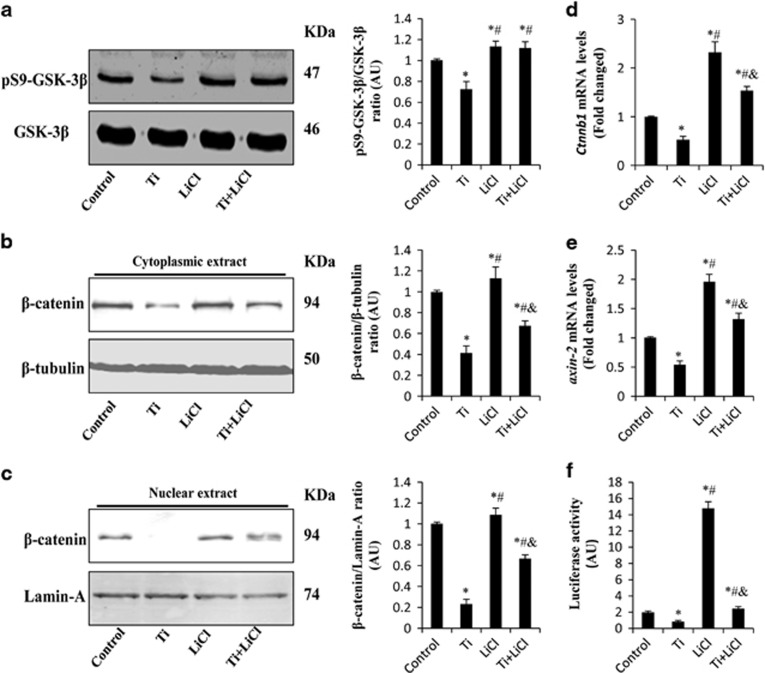
LiCl inhibits GSK-3*β* activation and upregulates *β*-catenin expression in Ti particle-stimulated MC3T3-E1 cells. MC3T3-E1 cells were pretreated with 10 mM LiCl for 2 h, and then cultured in osteogenic medium containing 0.1 mg/ml Ti particles for 24 h. (**a**) pSer9-GSK-3*β* and GSK-3*β* were determined by western blot. (**b** and **c**) Cytoplasmic and nuclear extracts were isolated and analyzed by western blot analysis using *β*-catenin antibody. (**d** and **e**) Gene expression of *ctnnb1* and *axin-2* was identified using RT-PCR. (**f**) Luciferase activity was measured in cell lysates. *P<0.05 *versus* control group; ^#^*P*<0.05 *versus* Ti group; ^&^*P*<0.05 *versus* LiCl group

**Figure 4:**
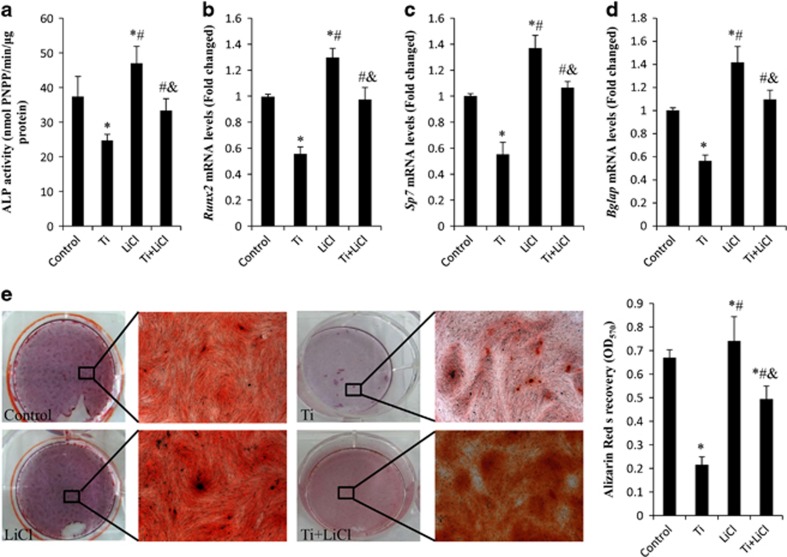
LiCl increases the differentiation potential of Ti particle-stimulated osteoblast cells. MC3T3-E1 cells were pretreated with 10 mM LiCl for 2 h, and then cultured in osteogenic medium containing 0.1 mg/ml Ti particles for the indicates times. (**a**) ALP activity, and (**b**) *runx2* and (**c**) *sp7* mRNA levels were determined after 3 days of incubation. (**d**) Gene expression levels of *bgalp* were determined on day 10. (**e**) Matrix mineralization of differentiated MC3T3-E1 cells assessed by ARS staining on day 21. *P<0.05 *versus* control group; ^#^*P*<0.05 *versus* Ti group; ^&^*P*<0.05 *versus* LiCl group

To investigate whether the Ti-induced modulation of GSK-3*β/β*-catenin signaling pathway was associated with its inhibition of osteoblast differentiation, MC3T3-E1 cells were pretreated with LiCl (GSK-3*β* inhibitor, 10 mM), and then stimulated with Ti particles for different durations. As shown in Figure 3, LiCl increased pSer9-GSK-3*β* expression, promoted *β*-catenin nuclear translocation and further upregulated *β*-catenin signal activity even in the presence of Ti particles. After co-culture with LiCl for 3 days, ALP activity was 32.4±2.4 nmol pNPP/min/*μ*g protein, which was significantly higher than in cells treated with Ti particles alone (24.7±1.8 nmol pNPP/min/*μ*g protein). LiCl treatment markedly raised the mRNA levels of osteoblastogenesis-related genes, including *runx2*, *sp7* and *bgalp*, compared with the group treated with Ti particles alone. Moreover, LiCl stimulated cell mineralization by approximately 122.7% when compared with the Ti particle-treated group (Figure 4). These data demonstrated that the GSK-3*β* inhibitor promoted Ti particle-induced osteoblast differentiation.

### Blocking *β*-catenin signal attenuated the protective effects of GSK-3*β* inhibitors on osteoblast differentiation

**Figure 5:**
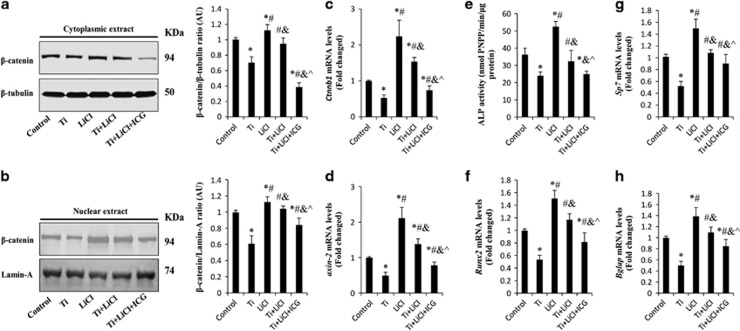
ICG-001 attenuates the protective effects of LiCl on the osteogenic activity of MC3T3-E1 cells stimulated with Ti particles. MC3T3-E1 cells were pretreated with 10 *μ*m ICG-001 for 30 min; then, 10 mM LiCl and 0.1 mg/ml Ti particles were added and cultured in osteogenic medium for the indicated times. (**a** and **b**) Cytoplasmic and nuclear extracts were isolated and analyzed by western blot analysis using *β*-catenin antibody. (**c**) *ctnnb1* and (**d**) *axin-2* mRNA levels were identified using RT-PCR after 24 h of incubation. (**e**) ALP activity, and (**f**) *runx2* and (**g**) *sp7* mRNA levels were determined after 3 days of incubation. (**h**) Gene expression levels of *bgalp* were determined on day 10. *P<0.05 *versus* control group; ^#^*P*<0.05 *versus* Ti group; ^&^*P*<0.05 *versus* LiCl group; ^*P*<0.05 *versus* Ti+LiCl group

To further confirm that the GSK-3*β* inhibitor promoted Ti particle-induced osteoblast differentiation via the *β*-catenin signal pathway, MC3T3-E1 cells were exposed to ICG-001 (a selective inhibitor of *β*-catenin, 10 *μ*m) for 30 min, and then stimulated with LiCl and Ti particles for 24 h. Western blots demonstrated that LiCl significantly increased *β*-catenin accumulation in the nucleus. However, the positive effects of LiCl on *β*-catenin accumulation, as well as *ctnnb1* and *axis-2* gene copies were significantly impaired by the addition of exogenous ICG-001 (Figures 5a and d). Moreover, after co-culture with ICG-001 for 3 days, ALP activity decreased to 26.7±4.9 nmol pNPP/min/*μ*g protein, which was significantly lower than that in the LiCl+Ti groups (32.3±6.3 nmol pNPP/min/*μ*g protein; Figure 5e). In addition, RT-PCR results showed that ICG-001 suppressed the gene expression of *runx2*, *sp7* and *bgalp*, indicating that the GSK-3*β* inhibitor-induced cell differentiation was mediated by the *β*-catenin signal pathway (Figures 5f–h).

### GSK-3*β/β*-catenin signaling pathway and RANKL/OPG ratio in Ti particle-stressed osteoblast cells

**Figure 6:**
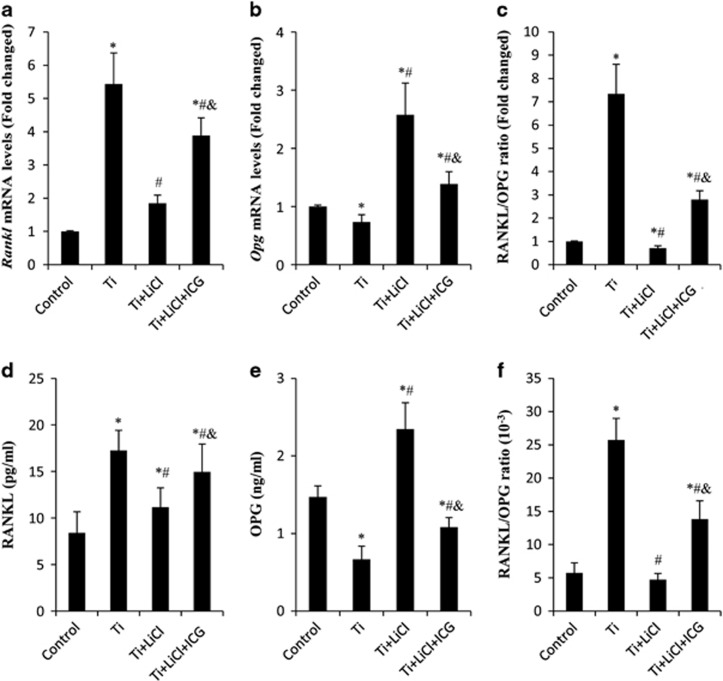
Ti particles regulate RANKL and OPG expression via GSK-3*β* and *β*-catenin signal pathway. MC3T3-E1 cells were pretreated with 10 *μ*m ICG-001 for 30 min; then, 10 mM LiCl and 0.1 mg/ml Ti particles were added and cultured in the osteogenic medium for 24 h. (**a**) *rankl* and (**b**) *opg* mRNA levels in MC3T3-E1 cells were evaluated using RT-PCR. (**c**) Ratio of RANKL/OPG mRNA. (**d**) RANKL and (**e**) OPG protein levels in MC3T3-E1 culture medium were identified by ELISA. (**f**) Ratio of RANKL/OPG protein levels. *P<0.05 *versus* control group; ^#^*P*<0.05 *versus* Ti group;^&^*P*<0.05 *versus* LiCl group; ^*P*<0.05 *versus* Ti+LiCl group

As shown in Figure 6, after stimulation with Ti particles for 24 h, the gene expression of *RANKL* increased 4.7-fold; however, the gene expression of *OPG* decreased by 27% compared with the control group. Thus, the RANKL/OPG ratio increased 7.3-fold. When GSK-3*β* activity was downregulated, the mRNA levels of *RANKL* were markedly reduced; in contrast, there was an increase in *OPG* mRNA levels compared with that in Ti particle-treated group. In addition, RANKL/OPG ratio was reduced after the addition of the GSK-3*β* inhibitor. Secreted RANKL and OPG in the culture supernatants of MC3T3-E1 were measured using ELISA. Results showed that the levels of RANKL and OPG were 17.36±2.28 pg/ml and 0.67±0.17 ng/ml, respectively, in the Ti particle-treated groups; however, with LiCl treatment, the secretion of RANKL (11.24±2.16 pg/ml) was reduced, whereas the secretion of OPG (2.45±0.32 ng/ml) was increased. Consequently, the mean RANKL/OPG ratio decreased from (25.75±3.21)/10^3^ (Ti group) to (4.76±0.89)/10^3^ (Ti+LiCl group). In addition, pretreatment with ICG-001 reversed the effects of the GSK-3*β* inhibitor.

### GSK-3*β/β*-catenin signal pathway and Ti particle-induced bone destruction

**Figure 7:**
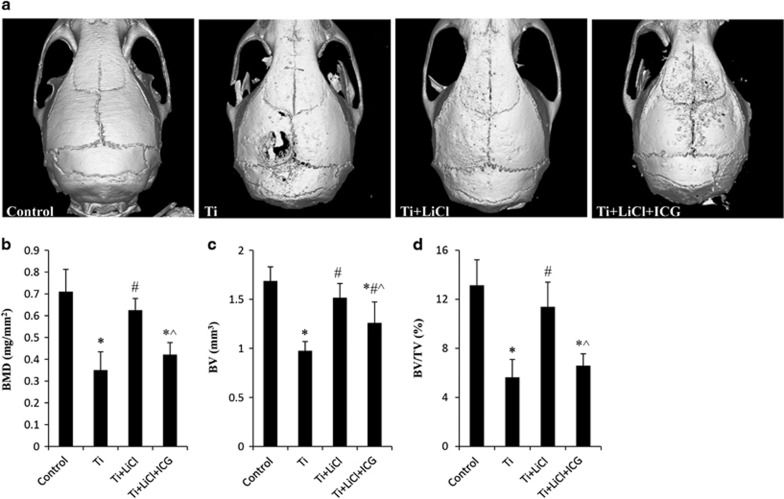
Ti particle-induced osteolysis through modulating GSK-3*β/β*-catenin signal pathway in a murine calvariae model. (**a**) Representative micro-CT reconstruction of calvariae in each group. (**b**) BMD, (**c**) BV and (**d**) BV/TV within the ROI were determined. *P<0.05 *versus* control group; ^#^*P*<0.05 *versus* Ti group; ^&^*P*<0.05 *versus* LiCl group; ^*P*<0.05 *versus* Ti+LiCl group

**Figure 8:**
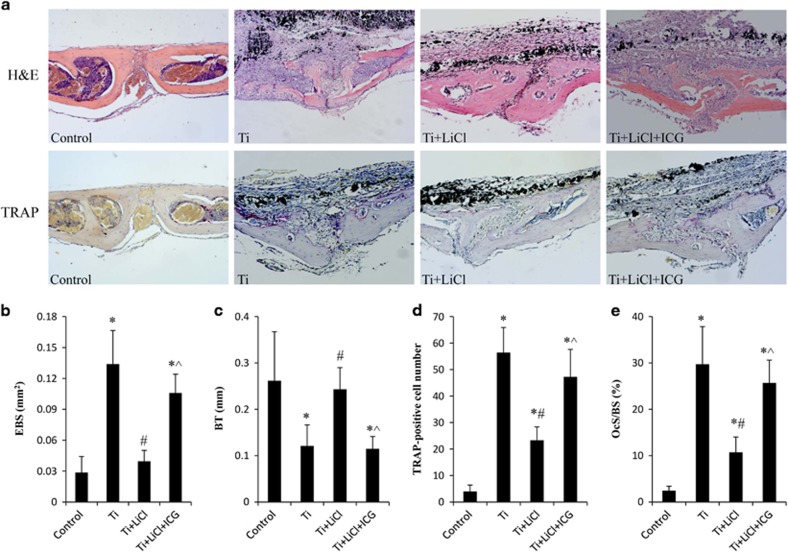
Histological analysis of bone destruction in the calvariae of mice. (**a**) Representative H&E and TRAP images in calvariae of mice. (**b**) EBS (mm^2^), (**c**) BT (mm), (**d**) number of TRAP-positive cells and (**e**) OCS/BS within the ROI were identified. *P<0.05 *versus* control group; ^#^*P*<0.05 *versus* Ti group; ^&^*P*<0.05 *versus* LiCl group; ^*P*<0.05 *versus* Ti+LiCl group

To investigate whether Ti particles induced bone loss via modulating the GSK-3*β/β*-catenin signal pathway *in vivo*, the murine calvarial model was used to mimic the molecular pathogenesis of peri-prosthetic osteolysis. Daily injection of the GSK-3*β* inhibitor evidently increased bone mineral density (BMD) within a region of interest (ROI) of calvaria exposed to Ti particles (0.63±0.05 mg/mm^2^ *versus* 0.35±0.08 mg/mm^2^, *P*<0.05, *n*=5). Micro-CT also revealed a marked increase in bone volume (BV) and BV against tissue volume (BV/TV) ratio in mice stimulated with the GSK-3*β* inhibitor. In ICG-001-treated mice, BMD, BV and BV/TV were markedly decreased compared with mice stimulated with GSK-3*β* inhibitor (Figure 7). Consistent with the results of micro-CT, H&E staining showed that treatment with the GSK-3*β* inhibitor decreased the area of eroded bone surface (EBS) and increased bone thickness (BT) within an ROI in the calvaria of mice in the Ti particle-stimulated group. Tartrate-resistant acid phosphatase (TRAP) staining revealed the presence of lots of TRAP-positive cells within ROI in Ti particle-treated group. When LiCl was administered, TRAP-positive cell numbers decreased by 58.7%, and osteoclast surface per bone surface (OCS/BS) decreased by 64.0%, compared with those in Ti particle-treated mice. However, local treatment with the *β*-catenin selective inhibitor reversed the protective effects of LiCl on bone destruction (Figure 8), suggesting that Ti particles induced bone loss via the regulation of GSK-3*β/β*-catenin signal pathway.

## Discussion

Wear debris from prosthesis wear are critical for the initiation of aseptic loosening. To understand the biology between wear debris and aseptic loosening, we and others previously investigated how wear debris affects osteoclast stimulation.^17, 18^ However, particle exposure not only has a direct effect on bone homeostasis by stimulating osteoclastogenesis, but also has adverse effects on osteoblastogenesis.^19^ Recent studies demonstrated that marked impairment of bone formation from reduced osteoblast activity was a common feature of peri-prosthesis in wear particle-induced osteolytic bone disease.^19, 20^ However, the mechanism underlying the particle-induced inhibition on osteoblastogenesis is unclear. In this study, we demonstrated that Ti particles significantly impacted MC3T3-E1 cell differentiation and induced bone destruction in the calvarial osteolysis model. These effects were affected by the activation of GSK-3*β* and further suppression of *β*-catenin signal pathway. These findings supported the concept that GSK-3*β/β*-catenin pathway has an importnat role in the development of aseptic loosening.

Osteoblasts, derived from the mesenchymal stem cells, are the main cells in bone tissue and bone formation, and are critical for prosthesis stability.^2, 5^ Continuous exposure to wear debris, including polyethylene, Ti and polymethyl methacrylate, compromises the function of mature osteoblasts, as well as suppresses bone formation and differentiation of osteoblast precursors,^5, 6, 20, 21^ which is in line with the results in this study. It is well-known that bone formation is impaired in aseptic loosening, which in part causes a net bone loss. The GSK-3*β/β*-catenin signal pathway has been shown to regulate osteoblast differentiation and bone formation *in vivo*,^22^ and multiple studies have shown evidence for this pathway in the initiation of osteolytic disease.^16, 23^ In support of this, we demonstrated a clear decrease in pSer9-GSK-3*β* and *β*-catenin expression in osteoblasts after stimulation with Ti particles, indicating that they decreased osteoblastogenesis by modulating the GSK-3*β/β*-catenin signal pathway in osteoblasts. In addition, we demonstrated that inhibition of the GSK-3*β* activity reversed the effects of Ti particles on osteoblasts differentiation, and reconstituted bone mass in calvariae stimulated with Ti particles.

GSK-3*β* is a critical regulator for normal bone mass. In this article, we provided evidence for GSK-3*β* mediating the reduction of bone formation in osteoblast progenitors treated with Ti particles. The decrease in osteoblast-related genes, including *runx2*, *sp7* and *bgalp*, supported the reduction in osteoblast activity in cells after activating GSK-3*β*. Previous studies demonstrated that GSK-3*β*^+/−^ mice have a higher bone formation rate, higher bone mass and more osteoblasts per bone surface.^12^ Furthermore, pharmacological inhibition of GSK-3*β* reduced ovariectomy-induced bone loss and enhanced BMD in wild-type animals.^24, 25^ In this study, the GSK-3*β* inhibitor increased baseline osteogenic activity, and restored the osteoblast differentiation capacity in Ti particle-stimulated cell cultures. Moreover, the GSK-3*β* inhibitor also increased BMD and BV/TV in calvariae of Ti particle-stimulated mice. These results suggested that loss of GSK-3*β* activity protects osteoblasts against Ti particle-induced deterioration. The involvement of the GSK-3*β* signal pathway in osteoblast activity loss is suggestive of the paradoxical phenomenon of Ti particle-induced bone destruction in aseptic loosening.

*β*-Catenin is a key factor of the canonical Wnt pathway activation, which is essential for proper bone development, and inhibition of this signal pathway downregulates bone formation.^13, 14^ In this study, the lesser nuclear translocation of *β*-catenin and reduction of TCF/LEF luciferase activity indicated the downregulation of the *β*-catenin signal pathway in Ti particle-stimulated MC3T3-E1 cells. Previous studies have demonstrated that *β*-catenin is essential for osteoblast differentiation, and its absence changed the differentiation potential of mesenchymal precursors from osteoblasts to chondrocytes.^26, 27^ Furthermore, GSK-3*β* regulates *β*-catenin activity through phosphorylation and degradation of *β*-catenin, and thus inhibits transcription of *β*-catenin responsive osteogenic genes.^13^ In this study, Ti particles suppressed phosphorylation at Ser9 in GSK-3*β*, and decreased the expression of *β*-catenin. In addition, the GSK-3*β* inhibitor promoted the expression of pSer9-GSK-3*β*, induced nuclear translocation of *β*-catenin and increased osteogenic activity of Ti particle-stressed cells, whereas administration of the *β*-catenin selective inhibitor reversed these effects. These results showed that Ti particles suppressed osteoblast differentiation and bone formation through the GSK-3*β/β*-catenin signal pathway.

Wear debris-induced osteolysis is the result of both increased osteoclast bone destruction and decreased osteoblast bone formation.^2, 5^ Indeed, Ti particles markedly increased the number of TRAP-positive cells in mice calvariae, which is consistent with previous studies.^3, 9, 10^ However, the number of TRAP-positive cells decreased in the group treated with the GSK-3*β* inhibitor compared with the Ti particle-treated group, thus indicating that GSK-3*β* is essential for osteoclast differentiation. A decrease in the RNAKL/OPG ratio, a critical regulator for osteoclast activation,^28^ in Ti particle-stressed osteoblasts may in part explain the reduction in osteoclast numbers in GSK-3*β* inhibitor-treated mice. Recently, Jiang *et al.*^29^ demonstrated that GSK-3*β* was inactivated during RANKL-induced osteoclast formation; however, whether GSK-3*β* regulates wear debris-induced osteoclastogenesis in a direct way remains unclear and requires further investigation.

In summary, this study showed that Ti particles may regulate osteoblast differentiation and bone formation through the GSK-3*β/β*-catenin signal pathway. Pharmacological inhibition of GSK-3*β* may impair Ti particle-induced inhibition of osteogenic activity, and reduce bone destruction in calvariae of mice stimulated with Ti particles. Collectively, our findings indicated that the GSK-3*β/β*-catenin signal pathway contributes to the osteogenic inhibition and bone destruction around loosening implants, and can be used as a potential therapeutic target for the treatment of peri-prosthetic osteolysis and aseptic loosening.

## Materials and methods

Animal experiments were performed in strict accordance with the principles and procedures of the National Institutes of Health (NIH) Guide for the Care and Use of Laboratory Animals and the Animal Care Committee of the First Affiliated Hospital of Soochow University. All experimental protocols in this study were approved by the ethics committee of the First Affiliated Hospital of Soochow University.

### Ti particle preparation

Ti particles were obtained from Alfa Aesar (catalog #00681, Tianjin, China) and were prepared as previously described.^3, 10, 16, 30^ Briefly, these particles were baked at 180 °C for 12 h, and then immersed in ethanol (75%) for 2 days. Endotoxin levels in the particles were determined using a commercial detection kit (Biowhittaker, Walkersville, MD, USA), and particles with <0.02 EU/ml endotoxin were used.

### Cell culture and differentiation

The murine MC3T3-E1 osteoblast cell line was obtained from ATCC (Manassas, MD, USA) and maintained in alpha-minimum essential medium (*α*-MEM) supplemented with 10% fetal bovine serum (FBS), 100 U/ml penicillin, 100 *μ*g/ml streptomycin and 2 mM l-glutamine. For differentiation experiments, cells were cultured in the above *α*-MEM and 50 *μ*g/ml ascorbic acid (Sigma, St. Louis, MO, USA), 10 mM *β*-glycerophosphate (Sigma), Ti particles, 10 mM lithium chloride (LiCl; Sigma) or 10 *μ*m ICG-001 (Tocris Bioscience, Bristol, UK). The control cultures did not contain Ti particles.

### Cell viability: CCK-8 assay

MC3T3-E1 cells (10^4^/ml) were seeded on 96-well plates and allowed to attach for 12 h in *α*-MEM containing 10% FBS. Cells were then cultured with 0.1 mg/ml Ti particles for 24, 48 or 72 h. At each time point, cells were incubated with fresh serum-free medium containing the CCK-8 (Dojindo Lab, Tokyo, Japan) reagent for 2 h at 37 °C. The culture medium was replaced with equal volumes of DMSO to dissolve formazan crystals, and then shook at room temperature for 10 min. Absorbance at 450 nm was obtained by using a microplate reader. Each reaction had three replicates and all experiments were repeated at least three times.

### RNA extraction and RT-PCR

Total RNA was extracted from MC3T3-E1 cells using TRIzol Reagent (Invitrogen, Carlsbad, CA, USA). RNA integrity was determined by separating the RNA on an agarose, and quality was assessed by having the A260/A280 ratio cutoff higher than 1.7. Total RNA (2 *μ*g) was used to synthesize first-strand cDNA with an RT-PCR kit (Invitrogen). Glyceraldehyde 3-phosphate dehydrogenase (*gapdh*) was used as an internal control. The mRNA levels of each gene from each experimental sample were normalized by comparison with a corresponding internal control using previously described methods.^10, 31^ The primers used in this study are shown in Table 1.

### ALP activity

ALP activity was determined using a p-nitrophenyl phosphate assay (pNPP; Sigma). Briefly, the cell layers were washed in ice-cold PBS to remove residual particles and detached cells. Then, the cells were lysed in a solution containing 10 mM Tris and 0.1% TritonX-100 (pH 7.5). After centrifugation, the supernatants were collected and incubated in an alkaline buffer (pH 10.5) containing 5 mM pNPP (substrate) and 2 mM MgCl_2_. Then, ALP activity was determined using the methods previously described.^32, 33^

### Quantification of mineralization

ARS was used to quantify the mineralization of MC3T3-E1 cultures in response to Ti particles and/or LiCl. Briefly, MC3T3-E1 cells were grown to confluence in culture media as previously described, and then grown for an additional 21 days in mineralization media in the presence or absence of Ti particles, and/or LiCl, at the indicated concentrations. Following treatment, mineralized osteoblasts were washed three times with PBS, and fixed in 4% paraformaldehyde for 1 h. The fixed layers were washed three times with excess dH_2_O before the addition of 40 mM ARS solution (pH 4.2; Sigma) for 30 min at room temperature with gentle agitation. The stained cells were observed through a microscope. To quantify mineralization, the stained layers were washed five times for 5 min each with excess dH_2_O, and three times with PBS for 5 min each at room temperature. Mineral bound ARS was solubilized by the addition of 10% cetylpyridinium chloride (pH 7.0; Sigma). Dye absorbance was measured at 570 nm. Six wells were analyzed per experiment. Experiments were repeated at least three times.

### Western blotting

Protein quantification was performed using the BCA protein assay reagent, and 20 *μ*g of nuclear or total cell protein was used for western blotting. Samples were separated on SDS-PAGE, and proteins were then transferred to PVDF membranes. Immunoblotting was performed as described previously using specific antibodies raised against *β*-catenin (Abcam, Shanghai, China; 1 : 10 000), GSK-3*β* (Abcam, 1 : 10 000) and pSer9-GSK-3*β* (Abcam, 1 : 5000). Protein quantification was performed using the Odyssey IR software, version 1.2 (LI-COR, Lincoln, NE, USA). Relative protein levels were calculated as the ratio of treated *versus* control, after normalizing against the housekeeping protein. Results are representative of three independent experiments.

### Luciferase reporter assay

To assay for the activation of *β*-catenin/TCF target genes, MC3T3-E1 cells were transfected transiently with 2 *μ*g TopFlash constructs by electroporation using Nucleofector technology. These constructs contained a firefly luciferase reporter under the control of a wild-type TCF/LEF-binding site. Cells were co-transfected with 0.2 *μ*g of *β*-galactosidase reporter vector as an internal standard for transfection. Twenty-four hours post-transfection, cells were stimulated with the indicated concentrations of Ti particles, LiCl and/or Wnt3a. Following 12 h of stimulation in *α*-MEM with 5% FBS, luciferase and *β*-galactosidase reporter activities were assayed using Bright Glo and *β*-Glo assay kits, respectively (Promega, Sunnyvale, CA, USA). Topflash results were normalized to the activity of *β*-galactosidase.

### ELISA

MC3T3-E1 cells with or without LiCl or ICG-001 pretreatment were incubated with 0.1 mg/ml Ti particles at 37 °C for 24 h. The culture medium was harvested for ELISA analysis. Mouse RANKL and OPG ELISA kits (R&D Systems, Shanghai, China) were used to determine the levels of the two cytokines according to the manufacture’s instruction.

### Animal studies

For this study, we established a murine calvariae osteolysis model was established. Briefly, 40 C57BL/6 female mice (8-weeks-old) were divided into four groups: (a) control; (b) Ti; (c) LiCl; and (d) LiCl and ICG-001. The mice were first anesthetized, and then Ti particles (20 mg) were placed on the surface of calvariae of mice in groups (b), (c) and (d) groups. After surgery, mice in groups (c) and (d) were gavage-fed 200 mg/kg LiCl every day. In addition, the LiCl-treated mice were injected with 10 *μ*l PBS or ICG-001 (10 *μ*g) at the surgery site before particle implantation, and then daily. After 2 weeks, mice were killed, and calvariae were collected for further analysis.

### Micro-CT analysis

The calvariae (*n*=5 per group) were scanned and reconstructed into a 3D structure with micro-CT (SkyScan1176, SkyScan, Aartselaar, Belgium) at a normal resolution of 18 *μ*m. The X-ray source was set at 80 kV and 100 *μ*A, and exposure time was 100 ms. X-ray projections were obtained at 0.9 intervals with a scanning angular rotation of 360°. A cylindrical ROI (3 × 3 × 1 mm) was identified to reduce the bias of quantitative analysis of the calvaria.^34^ Parameters including BMD (mg/mm^2^), BV (mm^3^) and BV/TV (%) were calculated using the Skyscan CT-analyzer software.

### Histological analysis

After decalcification in 10% EDTA for 21 days, calvariae (*n*=5 per group) were embedded in paraffin, and five sections were prepared for H&E and TRAP staining. EBS and BT were quantified from five consecutive sections per animal using Image Pro-Plus 6.0. (Media Cybernetics, Bethesda, MD, USA)^16, 35^ Then, TRAP-positive cells were identified, and OCS/BS (%) was calculated according to the methods established by Sawyer *et al.*^36^

### Statistical analysis

Data were obtained from three or more independent experiments. The significance of difference between groups was assessed by one-way ANOVA with post-hoc Tukey for multiple comparisons. Statistical significance was set as *P*-value <0.05.

## Figures and Tables

**Table 1:** Primers used for RT-PCR

**Genes**	**Sequence (5’-3’)**	
*Bgalp*	F:TCCCACACAGCAGCTTGGCCC	R: TGAGGCTCCAAGGTAGCGCCG
*Runx2*	F:TTGACCTTTGTCCCAATGC	R: AGGTTGGAGGCACACATAGG
*Sp7*	F:TGAGCTGGAACGTCACGTGC	R: AAGAGGAGGCCAGCCAGACA
*Ctnnb1*	F:ACGGTGCCGCGCCGCTTATA	R: TAGCCATTGTCCACGCAGCGG
*Axin-2*	F: GTCTCTACCTCATTTCCCGAGAAC	R: CGAGATCAGCTCAGCTGCAA
*Opg*	F: TGAAGCACCGGAGCTGTCCCC	R: AGGCCAAATGTGCTGCAGTTCG
*Rankl*	F: TCCTGAGACTCCATGAAAACG	R: CCCACAATGTGTTGCAGTTC
*Gapdh*	F: GAGAAGGCTGGGGCTCATTT	R: CCAATATGATTCCACCCATG

Shown are the details of the primers used for RT-PCR, including forward (F) and reverse (R) sequences

## References

[bib1] Della Valle CJ, Mesko NW, Quigley L, Rosenberg AG, Jacobs JJ, Galante JO. Primary total hip arthroplasty with a porous-coated acetabular component. A concise follow-up, at a minimum of twenty years, of previous reports. J Bone Joint Surg Am 2009; 91: 1130–1135. 19411461 10.2106/JBJS.H.00168

[bib2] Athanasou NA. The pathobiology and pathology of aseptic implant failure. Bone Joint Res 2016; 5: 162–168. 27146314 10.1302/2046-3758.55.BJR-2016-0086 PMC4921050

[bib3] Zhu S, Hu X, Tao Y, Ping Z, Wang L, Shi J et al. Strontium inhibits titanium particle-induced osteoclast activation and chronic inflammation via suppression of NF-κB pathway. Sci Rep 2016; 6: 36251. 27796351 10.1038/srep36251 PMC5087084

[bib4] Wang Z, Liu N, Liu K, Zhou G, Gan J, Wang Z et al. Autophagy mediated CoCrMo particle-induced peri-implant osteolysis by promoting osteoblast apoptosis. Autophagy 2015; 11: 2358–2369. 26566231 10.1080/15548627.2015.1106779 PMC4835204

[bib5] Goodman SB, Ma T, Chiu R, Ramachandran R, Smith RL. Effects of orthopaedic wear particles on osteoprogenitor cells. Biomaterials 2006; 27: 6096–6101. 16949151 10.1016/j.biomaterials.2006.08.023

[bib6] Yao J, Cs-Szabó G, Jacobs JJ, Kuettner KE, Glant TT. Suppression of osteoblast function by titanium particles. J Bone Joint Surg Am 1997; 79: 107–112. 9010191 10.2106/00004623-199701000-00011

[bib7] Córdova LA, Trichet V, Escriou V, Rosset P, Amiaud J, Battaglia S et al. Inhibition of osteolysis and increase of bone formation after local administration of siRNA-targeting RANK in a polyethylene particle-induced osteolysis model. Acta Biomater 2015; 13: 150–158. 25462844 10.1016/j.actbio.2014.10.042

[bib8] Chen M, Chen PM, Dong QR, Huang Q, She C, Xu W. p38 Signaling in titanium particle-induced MMP-2 secretion and activation in differentiating MC3T3-E1 cells. J Biomed Mater Res A 2014; 102: 2824–2832. 24115593 10.1002/jbm.a.34956

[bib9] Mediero A, Ramkhelawon B, Wilder T, Purdue PE, Goldring SR, Dewan MZ et al. Netrin-1 is highly expressed and required in inflammatory infiltrates in wear particle-induced osteolysis. Ann Rheum Dis 2016; 75: 1706–1713. 26452536 10.1136/annrheumdis-2015-207593 PMC5349296

[bib10] Yang H, Xu Y, Zhu M, Gu Y, Zhang W, Shao H et al. Inhibition of titanium-particle-induced inflammatory osteolysis after local administration of dopamine and suppression of osteoclastogenesis via D2-like receptor signaling pathway. Biomaterials 2016; 80: 1–10. 26695376 10.1016/j.biomaterials.2015.11.046

[bib11] Liang MH, Chuang DM. Differential roles of glycogen synthase kinase-3 isoforms in the regulation of transcriptional activation. J Biol Chem 2006; 281: 30479–30484. 16912034 10.1074/jbc.M607468200

[bib12] Noh T, Gabet Y, Cogan J, Shi Y, Tank A, Sasaki T et al. Lef1 haploinsufficient mice display a low turnover and low bone mass phenotype in a gender- and age-specific manner. PLoS ONE 2009; 4: e5438. 19412553 10.1371/journal.pone.0005438 PMC2673053

[bib13] Gordon MD, Nusse R. Wnt signaling: multiple pathways, multiple receptors, and multiple transcription factors. J Biol Chem 2006; 281: 22429–22433. 16793760 10.1074/jbc.R600015200

[bib14] Zhong Z, Zylstra-Diegel CR, Schumacher CA, Baker JJ, Carpenter AC, Rao S et al. Wntless functions in mature osteoblasts to regulate bone mass. Proc Natl Acad Sci USA 2012; 109: E2197–E2204. 22745162 10.1073/pnas.1120407109 PMC3421196

[bib15] Vestergaard P, Rejnmark L, Mosekilde L. Reduced relative risk of fractures among users of lithium. Calcif Tissue Int 2005; 77: 1–8. 16007481 10.1007/s00223-004-0258-y

[bib16] Geng D, Wu J, Shao H, Zhu S, Wang Y, Zhang W et al. Pharmaceutical inhibition of glycogen synthetase kinase 3 beta suppresses wear debris-induced osteolysis. Biomaterials 2015; 69: 12–21. 26275858 10.1016/j.biomaterials.2015.07.061

[bib17] Geng DC, Zhu XS, Mao HQ, Meng B, Chen L, Yang HL et al. Protection against titanium particle-induced osteoclastogenesis by cyclooxygenase-2 selective inhibitor. J Biomed Mater Res A 2011; 99: 516–522. 21913318 10.1002/jbm.a.33197

[bib18] Liu X, Zhu S, Cui J, Shao H, Zhang W, Yang H et al. Strontium ranelate inhibits titanium-particle-induced osteolysis by restraining inflammatory osteoclastogenesis*in vivo*. Acta Biomater 2014; 10: 4912–4918. 25078426 10.1016/j.actbio.2014.07.025

[bib19] Lee SS, Sharma AR, Choi BS, Jung JS, Chang JD, Park S et al. The effect of TNFα secreted from macrophages activated by titanium particles on osteogenic activity regulated by WNT/BMP signaling in osteoprogenitor cells. Biomaterials 2012; 33: 4251–4263. 22436801 10.1016/j.biomaterials.2012.03.005

[bib20] Atkins GJ, Welldon KJ, Holding CA, Haynes DR, Howie DW, Findlay DM. The induction of a catabolic phenotype in human primary osteoblasts and osteocytes by polyethylene particles. Biomaterials 2009; 30: 3672–3681. 19349075 10.1016/j.biomaterials.2009.03.035

[bib21] Ma GK, Chiu R, Huang Z, Pearl J, Ma T, Smith RL et al. Polymethylmethacrylate particle exposure causes changes in p38 MAPK and TGF-beta signaling in differentiating MC3T3-E1 cells. J Biomed Mater Res A 2010; 94: 234–240. 20166219 10.1002/jbm.a.32686

[bib22] Lin YY, Chen CY, Chuang TY, Lin Y, Liu HY, Mersmann HJ et al. Adiponectin receptor 1 regulates bone formation and osteoblast differentiation by GSK-3β/β-catenin signaling in mice. Bone 2014; 64: 147–154. 24713193 10.1016/j.bone.2014.03.051

[bib23] Gunn WG, Krause U, Lee N, Gregory CA. Pharmaceutical inhibition of glycogen synthetase kinase-3β reduces multiple myeloma-induced bone disease in a novel murine plasmacytoma xenograft model. Blood 2011; 117: 1641–1651. 21123822 10.1182/blood-2010-09-308171 PMC3318776

[bib24] Loiselle AE, Lloyd SA, Paul EM, Lewis GS, Donahue HJ. Inhibition of GSK-3β rescues the impairments in bone formation and mechanical properties associated with fracture healing in osteoblast selective connexin 43 deficient mice. PLoS ONE 2013; 8: e81399. 24260576 10.1371/journal.pone.0081399 PMC3832658

[bib25] Clément-Lacroix P, Ai M, Morvan F, Roman-Roman S, Vayssière B, Belleville C et al. Lrp5-independent activation of Wnt signaling by lithium chloride increases bone formation and bone mass in mice. Proc Natl Acad Sci USA 2005; 102: 17406–17411. 16293698 10.1073/pnas.0505259102 PMC1297659

[bib26] Dabernat S, Secrest P, Peuchant E, Moreau-Gaudry F, Dubus P, Sarvetnick N. Lack of beta-catenin in early life induces abnormal glucose homeostasis in mice. Diabetologia 2009; 52: 1608–1617. 19513688 10.1007/s00125-009-1411-y PMC4288852

[bib27] Dao DY, Jonason JH, Zhang Y, Hsu W, Chen D, Hilton MJ et al. Cartilage-specific β-catenin signaling regulates chondrocyte maturation, generation of ossification centers, and perichondrial bone formation during skeletal development. J Bone Miner Res 2012; 27: 1680–1694. 22508079 10.1002/jbmr.1639 PMC3399946

[bib28] Boyle WJ, Simonet WS, Lacey DL. Osteoclast differentiation and activation. Nature 2003; 423: 337–342. 12748652 10.1038/nature01658

[bib29] Jang HD, Shin JH, Park DR, Hong JH, Yoon K, Ko R et al. Inactivation of glycogen synthase kinase-3β is required for osteoclast differentiation. J Biol Chem 2011; 286: 39043–39050. 21949120 10.1074/jbc.M111.256768 PMC3234729

[bib30] Bechtel CP, Gebhart JJ, Tatro JM, Kiss-Toth E, Wilkinson JM, Greenfield EM. Particle-induced osteolysis is mediated by TIRAP/Mal *in vitro* and *in vivo*: dependence on adherent pathogen-associated molecular patterns. J Bone Joint Surg Am 2016; 98: 285–294. 26888676 10.2106/JBJS.O.00736

[bib31] Tian B, Jiang T, Shao Z, Zhai Z, Li H, Fan Q et al. The prevention of titanium-particle-induced osteolysis by OA-14 through the suppression of the p38 signaling pathway and inhibition of osteoclastogenesis. Biomaterials 2014; 35: 8937–8950. 25086794 10.1016/j.biomaterials.2014.06.055

[bib32] Ormsby RT, Cantley M, Kogawa M, Solomon LB, Haynes DR, Findlay DM et al. Evidence that osteocyte perilacunar remodelling contributes to polyethylene wear particle induced osteolysis. Acta Biomater 2016; 33: 242–251. 26796208 10.1016/j.actbio.2016.01.016

[bib33] Jiang X, Sato T, Yao Z, Keeney M, Pajarinen J, Lin TH et al. Local delivery of mutant CCL2 protein-reduced orthopaedic implant wear particle-induced osteolysis and inflammation*in vivo*. J Orthop Res 2016; 34: 58–64. 26174978 10.1002/jor.22977 PMC4817847

[bib34] Wedemeyer C, Xu J, Neuerburg C, Landgraeber S, Malyar NM, von Knoch F et al. Particle-induced osteolysis in three-dimensional micro-computed tomography. Calcif Tissue Int 2007; 81: 394–402. 17952672 10.1007/s00223-007-9077-2

[bib35] Kauther MD, Neuerburg C, Wefelnberg F, Bachmann HS, Schlepper R, Hilken G et al. RANKL-associated suppression of particle-induced osteolysis in an aged model of calcitonin and α-CGRP deficiency. Biomaterials 2013; 34: 2911–2919. 23357366 10.1016/j.biomaterials.2013.01.034

[bib36] Sawyer A, Lott P, Titrud J, McDonald J. Quantification of tartrate resistant acid phosphatase distribution in mouse tibiae using image analysis. Biotech Histochem 2003; 78: 271–278. 14989645 10.1080/10520290310001646668

